# Physiological and genomic features of a novel violacein-producing bacterium isolated from surface seawater

**DOI:** 10.1371/journal.pone.0179997

**Published:** 2017-06-22

**Authors:** Yue-Hong Wu, Hong Cheng, Lin Xu, Xiong-Bin Jin, Chun-Sheng Wang, Xue-Wei Xu

**Affiliations:** Key Laboratory of Marine Ecosystem and Biogeochemistry, Second Institute of Oceanography, State Oceanic Administration, Hangzhou, P. R. China; Korea University, REPUBLIC OF KOREA

## Abstract

Strains JW1^T^ and JW3, isolated from surface seawater of the Arabian Sea, were subjected to polyphasic taxonomic analysis. Cells of both strains were Gram-stain-negative, aerobic, and rod-shaped. They formed violet pigment and produced violacein. On the basis of 16S rRNA gene sequence analysis, strains JW1^T^ and JW3 showed high 16S rRNA gene sequence similarity with *Pseudoalteromonas byunsanensis* JCM12483^T^ (98.2%), *P*. *shioyasakiensis* SE3^T^ (97.8%), *P*. *arabiensis* JCM 17292^T^ (97.3%), and *P*. *gelatinilytica* NH153^T^ (97.1%). The 16S rRNA gene sequence similarity between JW1^T^ and JW3 was 100%. Phylogenetic analyses revealed that both strains fell within the cluster of the genus *Pseudoalteromonas* and represented an independent lineage. The average nucleotide identity and *in silico* DNA-DNA hybridization values between JW1^T^ and type strains of the closely related *Pseudoalteromonas* species were 70.9–83.3% and 20.0–26.4%, respectively. The sole respiratory quinone in both strains is ubiquinone 8 (Q-8). The principal fatty acids are summed feature 3 (C_16:1_*ω*7*c* and/or iso-C_15:0_ 2OH), C_18:1_*ω*7*c*, and C_16:0_. The major polar lipids are phosphatidylethanolamine, phosphatidylglycerol, one unidentified glycolipid, one unidentified aminolipid, and one unidentified phospholipid. The DNA G+C content was 43.3 mol%. Differential phylogenetic distinctiveness, chemotaxonomic differences, and phenotypic properties indicated that strains JW1^T^ and JW3 could be differentiated from the *Pseudoalteromonas* species with validly published names. Therefore, it is proposed that strains JW1^T^ and JW3 represent a novel species of the genus *Pseudoalteromonas*, for which the name *Pseudoalteromonas amylolytica* sp. nov. (type strain, JW1^T^ = CGMCC 1.15681^T^ = KCTC 52406^T^ = MCCC 1K02162^T^) is proposed.

## Introduction

The genus *Pseudoalteromonas*, the type genus of the family *Pseudoalteromonadaceae* [1], was proposed by Gauthier *et al*. (1995) [2]. Initially, the genus *Pseudoalteromonas* was differentiated from the genus *Alteromonas* based on the phylogenetic analysis of 16S rRNA gene sequences [2]. Currently, the genus *Pseudoalteromonas* consists of 43 species with validly published names (http://www.bacterio.net/p/pseudoalteromonas.html). Members of the genus *Pseudoalteromonas* are widespread in nature and have a great adaptability to marine environments, such as coastal, open, and deep seawaters, sediments, marine invertebrates, fish, and algae [3]. The genus *Pseudoalteromonas* is Gram-negative, aerobic or facultatively anaerobic, and rod-shaped, it requires Na^+^ ions for growth, usually does not denitrify, and possesses ubiquinone-8 (Q8) as major respiratory quinone [3].

Some *Pseudoalteromonas* species produce a variety of primary and secondary metabolites, including antibiotics [2], exopolymers [4, 5], hydrolytic enzymes [6, 7], and pigments [2, 8]. Violacein is a natural indolocarbazole compound formed by condensation of two molecules of tryptophan [9]. It is a potential pharmaceutical agent owing to its extensive biological properties, such as antibacterial, antiviral, antioxidant, and antitumor activities [10]. *Pseudoalteromona*s *luteoviolacea* has been reported to produce violacein [11]. Here, we present a polyphasic study describing two novel violacein-producing strains, both of which were isolated from surface water of the Arabian Sea.

## Materials and methods

### Organisms and culture conditions

Strains JW1^T^ and JW3 were isolated from the surface seawater collected from the Arabian Sea (E67° N24°). The seawater samples were stored at 4°C until use. Natural seawater agar (pH 7.2–7.4) supplemented with 0.05% peptone (w/v; BD, Sparks, MD, USA) and 0.01% yeast extract (w/v; BD) was used for isolation. The seawater samples were diluted using the standard ten-fold dilution plating technique and spread on natural seawater agar. After ten days of aerobic incubation at 30°C, two violet colonies, designated as JW1^T^ and JW3, were picked from different samples and purified by repeated restreaking. The purity was confirmed by the uniformity of cell morphology. The reference strains *P*. *byunsanensis* JCM 12483^T^, *P*. *shioyasakiensis* JCM 18891^T^, and *P*. *arabiensis* JCM 17292^T^ were obtained from the JCM (Japan Collection of Microorganisms). The reference strain *P*. *gelatinilytica* NH153^T^ was available in our lab [12]. Unless otherwise stated, the two strains were routinely cultured in marine broth 2216 (MB; BD) or on marine agar 2216 (MA; BD) at 30°C and stored at –80°C with 30% (v/v) glycerol.

### 16S rRNA gene and genome sequence determination

The 16S rRNA gene was amplified and analyzed as described previously [13]. PCR products were cloned into the vector pMD 19-T (TaKaRa, Dalian, China) and then sequenced to determine the almost-complete sequence of the 16S rRNA gene. High-quality genomic DNA was extracted with the AxyPrep™ Bacterial Genomic DNA Miniprep Kit (Axygen Scientific, Inc., Union City, CA, USA). The genomes of strains JW1^T^, JW3, and *P*. *byunsanensis* JCM 12483^T^ were sequenced using the Solexa paired-end sequencing technology with the Illumina HiSeq 2000 platform (Anoroad Gene Technology Co. Ltd, Beijing, China). One paired-end library was constructed with 500-bp insert size. The sequencing generated approx. 1 Gb of clean data (approx. 500-fold genome coverage). *De novo* assembly of the reads was carried out using SOAPdenovo (version 2.0.1) [14]. Assembly *k*-mer was tested from 57 to 64 for seeking the optimal value, using the abyss-pe script. Assembly quality was estimated using MUMmer [15]. Completeness of the genome sequence was addressed using the bioinformatics tool CheckM (http://ecogenomics.github.io/CheckM/) [16]. The complete sequence of the 16S rRNA gene was annotated via the RNAmmer 1.2 Server [17] and was compared with related sequences of reference organisms using the EzTaxon-e service [18].

### Phylogenetic status

Phylogenetic analysis was carried out using ARB (release 6.0.2) [19] and the All-Species Living Tree Project database (LTPs123, September 2015) [20]. The 16S rRNA gene sequences of strains JW1^T^ and JW3 were aligned with SILVA Incremental Aligner (SINA, version 1.2.11) (http://www.arb-silva.de) [21]. The alignment sequences were imported into the LTPs database and implemented in ARB. On the basis of the obtained All-Species Living Tree and the EzTaxon-e results, 24 species were selected and sequence data were aligned with ClustalW [22]. Phylogenetic trees were reconstructed using the MEGA 5 program package [23], using *Algicola sagamiensis* B-10-31^T^ as the outgroup, by neighbor-joining [24], maximum-parsimony [25], and maximum-likelihood methods [26]. Tree topology was evaluated by bootstrap analysis using 1000 resample datasets. Kimura two-parameter model [27] was used to calculate evolutionary distances and reconstruct phylogeny (neighbor-joining and maximum-likelihood methods).

The genomes of 20 type strains of *Pseudoalteromonas* species were retrieved from the GenBank database (S1 Fig). Six housekeeping genes, *atpD* (beta subunit for ATP synthase), *gyrB* (DNA gyrase beta subunit), *mreB* (rod shape-determining protein), *recA* (RNA recombinase alpha subunit), *rpoD* (RNA polymerase), and *topA* (DNA topoisomerase I) were used for multilocus sequence analysis (MLSA). The concatenated sequence of six single genes was obtained from the genome and subjected to maximum-likelihood phylogenetic analyses [26] with MEGA 5.

### Phenotypic characteristics

Cell morphology, size, and motility were examined using confocal laser scanning microscopy (TCS SP5; Leica) and transmission electron microscopy (JEM-1230; JEOL). The hanging-drop method was used for motility testing. Cell morphology and ultrastructure were observed using transmission electron micrographs.

The growth at various temperatures (4, 15, 20, 28, 30, 37, 45, and 50°C) was tested in MB. The pH range for growth was determined in the range of 5.0–10.5 with interval of 0.5 in MB by adding MES (pH 5.0–6.0), PIPES (pH 6.5–7.0), Tricine (pH 7.5–8.5), and CAPSO (pH 9.0–10.5) at a final concentration of 50 mM. pH values changed only minimally after autoclaving. Growth at different concentrations of NaCl (0, 0.5, 1.0, 3.0, 5.0, 7.5, 10.0, and 15.0%, w/v) was investigated using NaCl-free MB (prepared according to the MB formula, but without NaCl). Sea-salt requirement for growth was measured in the PY medium (peptone 5.0 g, yeast extract 1.0 g and distilled water 1 L, pH 7.6) supplemented with sea salts (Sigma) at various concentrations (0, 0.5, 1.0, 2.0, 3.0, 4.0, 4.5, and 5.0%, w/v). Growth was measured at 590 nm (OD_590_) with a UV/visible spectrophotometer (Ultrospec 6300 pro; Amersham Biosciences). Upper and lower limits for growth were confirmed when no growth was observed after cultivation for one month. For anaerobic growth, strains were incubated in the AnaeroPack-MicroAero anaerobic system (Mitsubishi). Sodium nitrate (20 mM) or sodium nitrite (20 mM) was used as a potential electron acceptor.

Gram reaction, oxidase and catalase activities, and hydrolysis of starch and Tween-20, -40, and -80 were tested according to [28]. Violacein was extracted according to [29] and the absorbance of the violet pigment was monitored from 350 nm to 1000 nm using a UV/visible spectrophotometer (DU800, Beckman Coulter). The molecular weight of violacein was deduced by LC-MS analysis according to [29]. Chromatography was carried out on a Agilent 1200. Compound separation was achieved on an analytical column (Extend-C18, 3.5 μm, 2.1 × 100 mm; Agilent). Analysis of the pigment by eletrospray ionization mass spectrometry was conducted with Finnigan LCQ DECA XP MAX mass spectrometer (Thermo Electron Corp., USA). The flow rate of the pigment solution was 15 μL/min. The utilization of carbon substrates as sole carbon and energy sources was tested in BM [30] supplemented with filter-sterilized complex nutrients (yeast extract, peptone and tryptone, 0.2%, w/v), sugars (0.2%, w/v), alcohols (0.2%, w/v), organic acids (0.1%, w/v), or amino acids (0.1%, w/v). Yeast extract (0.01%, w/v) was added as a growth factor. Acid production was evaluated using marine oxidation-fermentation medium supplemented with 1% filter-sterilized sugars [31]. API 20NE and API 20E tests (bioMérieux) were used according to the manufacturer’s instructions to determine additional physiological and biochemical characteristics. Strips were inoculated with a heavy bacterial suspension (*MacFarland 5 standard*) in AUX medium supplemented with 2% (w/v) sea salts (Sigma) [32]. API 20 NE and API 20 E strips were read after 48 h. Sensitivity to antimicrobial agents was determined with a two-layer plate method according to Wu *et al*. (2015)[33]. Four reference strains, *P*. *byunsanensis* JCM 12483^T^, *P*. *shioyasakiensis* JCM 18891^T^, *P*. *arabiensis* JCM 17292^T^, and *P*. *gelatinilytica* NH153^T^ were used as controls in the above tests.

### Chemotaxonomic characteristics

The cellular fatty acids of strains JW1^T^, JW3, and the reference strains were determined under identical conditions in parallel. The quadrant streak method was used for inoculation and cellular fatty-acid methyl esters were obtained from cells grown on MA at 30°C for 16 h from quadrant 3 (late exponential phase). Whole cell fatty acids were analyzed using the Microbial Identification System (MIDI Inc.) according to the manufacturer’s instructions. Isoprenoid quinones were extracted and purified by two-dimensional thin-layer chromatography (TLC) and then analyzed by LC-MS (Agilent 1200 and Thermo Finnigan LCQ DECA XP MAX mass spectrometer) [34]. Total lipids were extracted and separated by two-dimensional TLC [35] on silica gel 60 F254 plates (Merck). Four types of spray reagent were used to detect the corresponding lipids, including molybdophosphoric acid for total lipids, *α*-naphthol reagent for glycolipids, ninhydrin reagent for lipids containing free aminolipids, and molybdenum blue for phosphorus-containing lipids [36].

### Average nucleotide identities and genome analysis

The average nucleotide identity (ANI) was calculated using the OrthoANIu algorithm by ChunLab's online Average Nucleotide Identity calculator [37]. *In silico* DNA-DNA hybridization (DDH) values were calculated by GGDC [38].

rRNA genes were identified using the RNAmmer 1.2 Server [17] and tRNA genes were searched with the tRNAscan-SE 2.0 online server [39]. Gene prediction and functional annotation were carried out using the Rapid Annotation using Subsystem Technology (RAST) server online [40]. Selected predicted genes were classified using RPSBLAST against the COG database [41]. Translated genes were assigned to KEGG pathway using KEGG Automatic Annotation Server (KAAS) with the BBH method [42, 43]. Orthologous cluster analyses were carried out using OrthoMCL [44]. Shared and unique orthologous clusters were established with in-house shell scripts. CRISPR structures in the genomes were predicted with the CRISPRFinder program online (http://crispr.i2bc.paris-saclay.fr/Server/).

## Results and discussion

### Phenotypic features

Strains JW1^T^ and JW3 were Gram-stain-negative, aerobic and rod-shaped (0.7–1.2 μm in width and 1.8–3.0 μm in length) (S2 Fig). Colonies were violet, circular, convex, smooth, and 1–2 mm in diameter after one day of incubation at 30°C on MA. Optimal growth occurred at 30°C and pH 7.5. UV-visible absorption spectra and LC-MS analysis of the pigment isolated from strains JW1^T^, JW3, and *P*. *byunsanensis* JCM 12483^T^ showed the presence of violacein. Maximal absorption of UV-visible light was at 575 nm (S3A Fig). Mass spectrometry of the pigment revealed a parent ion [M-H]^−^at m/z 342.1, which was identical to that of violacein (S3B Fig) [45]. Both strains were positive for catalase, oxidase, tryptophan deaminase, and Voges–Proskauer reaction, able to hydrolyze esculin, gelatin, starch, Tween-20, -40 and -80, susceptible to (μg per disc unless otherwise stated) chloramphenicol (30), erythromycin (10), gentamicin (10), kanamycin (30), mefoxin (30), neomycin (30), nitrofurantoin (300), norfloxacin (10), polymyxin B (300 IU), rifampicin (5), streptomycin (10), tetracycline (30), and vancomycin (30), and resistant to ampicillin (10), cefalexin (30), nystatin (100), and penicillin G (10 IU). Detailed phenotypic characteristics are given in the species description and Table 1.

**Table 1:** Differential phenotypic characteristics of strains JW1^T^, JW3, and closely related members of the genus *Pseudoalteromonas*. Strains/species: 1, strain JW1^T^; 2, strain JW3; 3, *P*. *byunsanensis* JCM 12483^T^; 4, *P*. *shioyasakiensis* JCM 18891^T^; 5, *P*. *arabiensis* JCM 17292^T^; 6, *P*. *gelatinilytica* NH153^T^. All data were obtained in this study and under identical conditions, unless indicated otherwise.

Characteristics	1	2	3	4	5	6
Color	violet	violet	violet*	non-pigmented^†^	non-pigmented^‡^	non-pigmented^§^
Violacein production	+	+	+	–	–	–
Growth in NaCl (%):						
Range	0.5–10	0.5–10	0.5–5*	0.5–12^†^	0.5–10^‡^	0–10^§^
Optimum	1–3	1–3	1.5–2*	1–3^†^	2–3^‡^	3–5^§^
Growth pH:						
Range	6–10.5	6–10.5	5–10*	5.5–9.5^†^	5.0–10.0^‡^	5.5–9.5^§^
Optimum	7.5	7.5	8.0*	6.5–8.0^†^	7.0–8.0^‡^	7.5–8.5^§^
Growth temperature (ºC):						
Range	20–40	20–40	10–40*	5–40^†^	6–35^‡^	15–45^§^
Optimum	30	30	25–30*	28–30^†^	25^‡^	37^§^
Arginine dihydrolase	–	–	–	+	+	–
Citrate utilization	–	–	–	+	+	+
Nitrate reduction	–	–	–	–	+	–
Tryptophan deaminase	+	+	+	–	–	–
Urease	–	–	–	–	+	+
Voges–Proskauer	+	+	+	+	–	+
**Hydrolysis of:**						
Starch	+	+	+	–	–	–
Tween-40	+	+	+	–	+	–
**Utilization of:**						
_L_-Arabinose	–	–	–	+	+	+
Cellobiose	–	+	+	–	+	+
Ethanol	–	–	–	+	+	+
_D_-Fructose	–	–	–	+	–	+
_D_-Mannitol	–	–	–	–	–	+
_D_-Mannose	–	–	–	+	+	+
Sucrose	+	+	–	+	+	+
_D_-Trehalose	+	+	+	+	–	+
**Acid production:**						
Cellobiose	–	+	+	–	+	–
Ethanol	–	–	–	–	+	–
_D_-fructose	–	–	–	+	–	–
_D_-Galactose	–	+	–	–	–	–
_D_-Glucose	+	+	+	–	–	+
_D_-Mannitol	–	–	–	–	–	+
_D_-Mannose	–	–	–	+	+	+
_L_-Rhamnose	–	+	–	–	–	–
Sucrose	+	+	–	+	+	–
_D_-Trehalose	+	+	+	+	–	+
**Susceptibility to:**						
Gentamicin (10 μg)	+	+	+	+	–	+
Nitrofurantoin (300 μg)	+	+	+	–	–	–
Streptomycin (10 μg)	+	+	+	+	+	–
Tetracycline (30 μg)	+	+	+	–	–	+
**ANI value (%)**						
With JW1^T^	100	99.9	83.3	71.0	70.9	70.9
*in silico* DDH values						
With JW1^T^	100	99.9	26.4	20.0	20.1	20.0

*Data were taken from Park *et al*. (2005) [8]

^†^Data were taken from Matsuyama *et al*. (2014) [4]

^‡^Data were taken from Matsuyama *et al*. (2013) [5]

^§^Data were taken from Yan *et al*. (2016) [12].

### 16S rRNA gene sequence similarities and phylogenetic analysis

The 16S rRNA gene sequences of strains JW1^T^ and JW3 (1527 nt) were obtained. According to EzTaxon as well as ClustalW results of 16S rRNA gene sequence comparison to representative bacteria with validly published names, strains JW1^T^ and JW3 showed high 16S rRNA gene sequence similarity to *P*. *byunsanensis* JCM12483^T^ (98.2%), *P*. *shioyasakiensis* SE3^T^ (97.8%), *P*. *arabiensis* JCM 17292^T^ (97.3%), and *P*. *gelatinilytica* NH153^T^ (97.1%), and exhibited less than 97.0% 16S rRNA gene sequence similarity with the type strains of other *Pseudoalteromonas* species. The 16S rRNA gene sequence similarity between strains JW1^T^ and JW3 was 100%.

**Fig 1:**
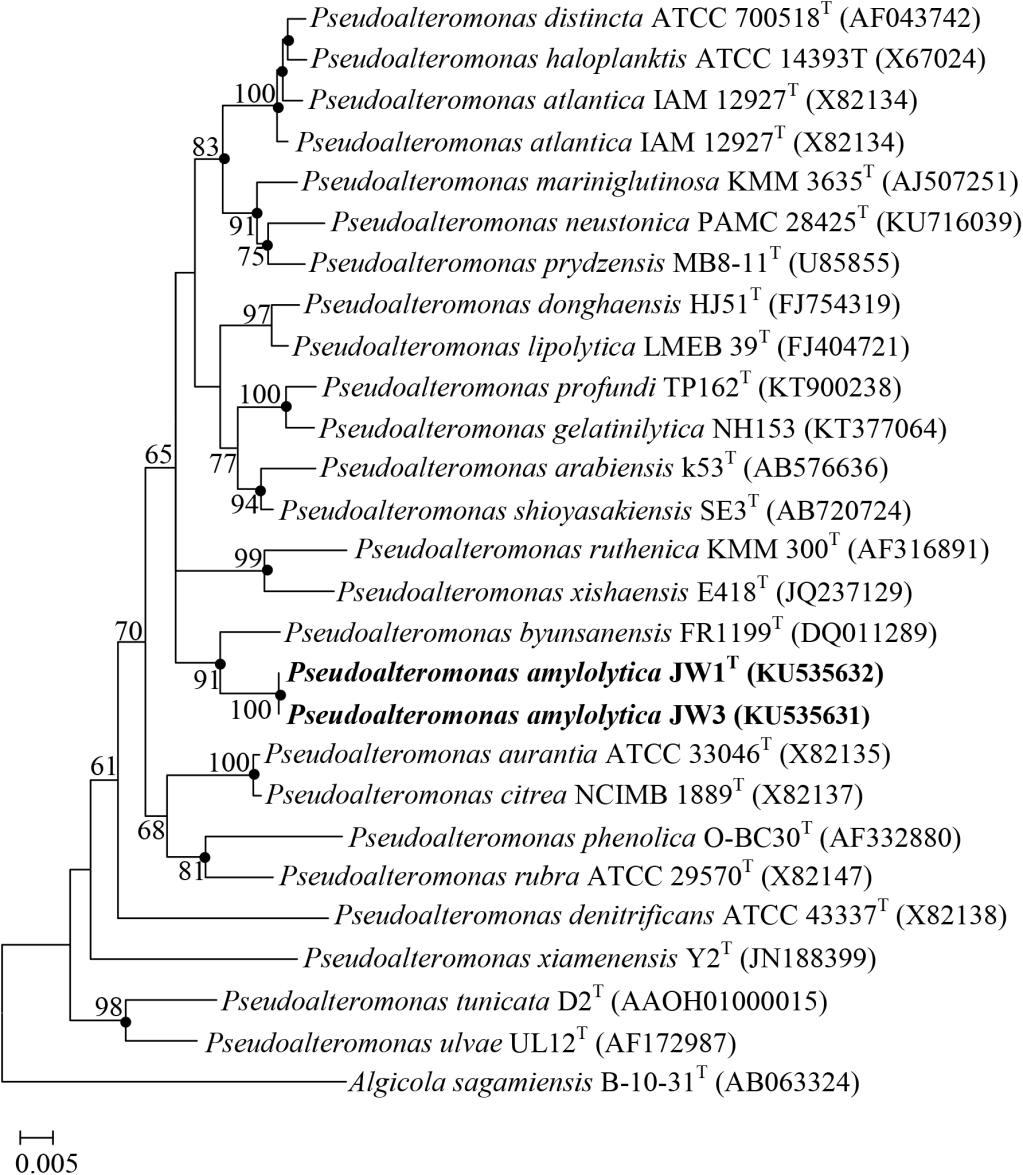
Neighbor-joining phylogenetic tree based on 16S rRNA gene sequences showing the phylogenetic relationships of JW1^T^, JW3, and related taxa. Bootstrap values (>60%) based on 1,000 replications are shown at branch nodes. Filled circles indicate that the corresponding nodes were also recovered in the trees generated with the maximum-likelihood and maximum-parsimony algorithms. Bar, 0.005 substitutions per nucleotide position.

The All-Species Living Tree indicated that the genus *Pseudoalteromonas* forms a monophyletic clade and strains JW1^T^ and JW3 fall within the cluster comprising the *Pseudoalteromonas* species. The topologies of neighbor-joining, maximum-likelihood, and maximum-parsimony phylogenetic trees based on the 16S rRNA gene also supported the notion that strains JW1^T^ and JW3 formed a stable lineage, with a high bootstrap value of 100%, and a distinct lineage from *P*. *byunsanensis* (bootstrap value 91%) (Fig 1). Phylogenetic trees based on concatenated sequences of the six housekeeping genes *atpD*, *gyrB*, *mreB*, *recA*, *rpoD*, and *topA* confirmed that the two strains formed a clade with *P*. *byunsanensis* as well as *P*. *citrea* and could not be associated with any of the recognized species in the genus *Pseudoalteromonas* (S1 Fig). Phylogenetic analysis indicated JW1^T^ and JW3 form another, independent lineage and might represent a novel member of the genus *Pseudoalteromonas*.

### Chemotaxonomic analysis

Chemotaxonomic data supported the results of the phylogenetic analysis. The sole respiratory quinone found in strains JW1^T^ and JW3 was Q8, in line with all members of the genus *Pseudoalteromonas* [3]. Fatty-acid analysis revealed that summed feature 3, C_18:1_*ω*7*c*, and C_16:0_ were the major fatty acids in JW1^T^ and JW3 and the references strains (Table 2). JW1^T^ and JW3 possessed phosphatidylethanolamine and phosphatidylglycerol as the major polar lipids, similar to the reference strains. In addition, JW1^T^ and JW3 possessed three unidentified glycolipids (GL2–GL4), one unidentified aminolipid (AL2), and one unidentified phospholipid (PL1) as moderate-to-minor polar lipids, which were similar to those of *P*. *byunsanensis* JCM 12483^T^ (S4 Fig). Moreover, JW1^T^ and JW3 possessed aminolipid, glycolipid, and phospholipid, all of which were detected in the four reference strains (S4 Fig) [12].

**Table 2:** Fatty acid compositions (%) of JW1^T^, JW3, and related *Pseudoalteromonas* species. Strains/species: 1, isolate JW1^T^; 2, isolate JW3; 3, *P*. *byunsanensis* JCM 12483^T^; 4, *P*. *shioyasakiensis* JCM 18891^T^; 5, *P*. *arabiensis* JCM 17292^T^; 6, *P*. *gelatinilytica* NH153^T^. All data were taken from this study. Fatty acids representing less than 0.1% in all strains were omitted.–, Not detected; tr, traces (<1.0%).

Fatty acid	1	2	3	4	5	6
**Straight-chain**						
C_10:0_	1.7	1.7	tr	–	–	–
C_12:0_	1.1	1.4	1.2	2.4	3.5	1.9
C_14:0_	tr	1.1	tr	1.1	1.6	1.0
C_16:0_	18.4	16.5	21.0	22.7	27.1	23.3
C_18:0_	1.6	1.0	1.8	5.1	3.0	3.7
**Unsaturated**						
C_15:1_*ω*8*c*	–	tr	1.0	–	tr	tr
C_17:1_*ω*8*c*	tr	tr	tr	tr	tr	1.2
C_18:1_ω6c	4.1	4.6	–	–	–	–
C_18:1_*ω*7*c*	18.4	13.9	21.2	20.4	14.6	22.6
C_18:1_*ω*9*c*	tr	tr	tr	tr	tr	1.2
**Hydroxy**						
C_10:0_ 3OH	6.3	7.9	7.3	1.0	tr	1.0
C_12:0_ 3OH	7.2	9.3	6.2	10.4	9.8	7.6
Summed feature*						
3	29.9	31.1	29.3	27.5	30.9	27.4
**Unknown**						
11.799	5.5	6.2	5.7	1.9	tr	1.8

*Summed features represent groups of two fatty acids that could not be separated by GLC with the MIDI system. Summed feature 2 contained C_14:0_ 3OH and/or iso-C_16:1_ I; Summed feature 3 contained C_16:1_*ω7c* and/or iso-C_15:0_ 2OH.

Chemotaxonomic data of JW1^T^, JW3, and their relatives also showed some clear differences in fatty acid composition and polar lipid profile. The percentage of C_16:0_ of strains JW1^T^ and JW3 (18.4% and 16.5%, respectively) was lower than that of the reference strains (21.0–27.1%). JW1^T^ and JW3 contained C_18:1_*ω*6c (4.1% and 4.6%, respectively), which were not detected in the reference strains (Table 2**)**. One unidentified aminolipid (AL1) was present in both strain JW1^T^ and JW3, but was not detected in the *P*. *byunsanensis* JCM 12483^T^. *P*. *byunsanensis* JCM 1248 possessed three unidentified aminolipids (AL3–AL5) and three unidentified lipids (L3–L5), while strains JW1^T^ and JW3 did not. Diphosphatidylglycerol was present in *P*. *shioyasakiensis* JCM 18891^T^, *P*. *arabiensis* JCM 17292^T^, and *P*. *gelatinilytica* NH153^T^ [12], but not in JW1^T^ and JW3 (S4 Fig).

### *In silico* DNA-DNA relatedness

The DNA G+C content of strains JW1^T^ and JW3 calculated from the genome sequence was 43.3 mol%, a value in the range reported for members of the genus *Pseudoalteromonas*, i.e. 38–48 mol% [46]. JW1^T^ and the reference strains exhibited ANI values of 70.9–83.3% (Table 1). These ANI values were far below the threshold of species boundary (94–96%) [47], indicating low taxonomic relatedness between JW1^T^ and the reference strains. The recommended results (formula 2) of the *in silico* DDH analysis revealed that JW1^T^ and the reference strains shared 20.0–26.4% DNA relatedness (Table 1). The values were below 70%, indicating that the strains should be assigned to different genomic species [48]. In addition, the ANI and *in silico* DDH values between JW1^T^ and JW3 were 99.9%. These values were above the species boundary (94–96% for ANI values and 70% for *in silico* DDH values), suggesting that JW1^T^ and JW3 represent an identical genospecies.

### Genomic features

General features of JW1^T^ and JW3 are displayed in Table 3 and S1 Table. The bioinformatics tool CheckM indicated that the genome completeness was 100% for both JW1^T^ and JW3, with a contamination percentage of 0.4% and 0.5%, respectively. Genome sequence completeness ≥95%, with ≤5% contamination, is considered to indicate an excellent reference genome for deeper analyses [16]. The genome size of the two strains and their related *Pseudoalteromonas* species varied from 4.5 Mb to 4.8 Mb. This variation can be attributed partially to the draft nature of the sequence. COG assignments were similar for all genomes. Twenty-two COG classes were annotated in the genomes of JW1^T^, JW3, *P*. *byunsanensis* JCM 12483^T^, and *P*. *arabiensis* JCM 17292^T^, and 23 COG classes (with an extra Cytoskeleton class) were detected in the genomes of *P*. *shioyasakiensis* JCM 18891^T^ and *P*. *gelatinilytica* NH153^T^ (S1 Table). Furthermore, 13 and 6 unique orthologous clusters were found in the genomes of JW1^T^ and JW3, respectively (Table 3). The two strains shared 640 unique orthologous clusters.

**Table 3:** Genome statistics of JW1^T^, JW3, and related *Pseudoalteromonas* species. Strains/species: 1, strain JW1^T^ (MKJU00000000); 2, strain JW3 (MKJT00000000); 3, *P*. *byunsanensis* JCM 12483^T^ (MNAN00000000); 4, *P*. *shioyasakiensis* JCM 18891^T^ (LRUE00000000); 5, *P*. *arabiensis* JCM 17292^T^ (LRUF00000000); 6, P. *gelatinilytica* NH153^T^ (LRRU00000000). +, Detected;–, not detected.

Genome characteristics	1	2	3	4	5	6
Size (Mbp)	4.86	4.85	4.74	4.81	4.46	4.8
G+C content (mol%)	43.3	43.3	42.5	41.3	40.9	41.4
Total number of genes	4212	4204	4096	4350	4302	4302
Protein coding sequences (CDS)	4035	4026	3919	4230	4184	4184
Pseudogene	61	58	54	25	16	16
rRNA genes	19	18	17	13	7	7
tRNA genes	95	98	97	78	94	91
CRISPRs structure	2	2	2	3	2	4
*vioABCDE* operon	+	+	+	–	–	–
Unique orthologous clusters	13	6	646	426	628	337

Genome analysis of JW1^T^, JW3, and the reference strains indicated the presence of genes encoding clustered regularly interspaced short palindromic repeats (CRISPRs; Table 3). CRISPRs, in association with CRISPR-associated (Cas) proteins, make up the immune system that confers resistance to foreign genetic elements, such as plasmids and phages [49]. The CRISPRs/Cas system is being used for gene editing. Colonies of JW1^T^ and JW3 were violet and the two strains produced violacein. The biosynthesis of violacein begins with _L_-tryptophan and is successively catalyzed by enzymes VioA, B, E, D, and C, all of which are encoded by the *vioABCDE* operon [50]. Strains JW1^T^, JW3, and *P*. *byunsanensis* JCM 12483^T^ produced violacein and possessed *vioABCDE* operon, while *P*. *arabiensis* JCM 17292^T^, *P*. *shioyasakiensis* JCM 18891^T^ and *P*. *gelatinilytica* NH153^T^ did not (Table 3 and S3 Fig).

The genomes of strains JW1^T^ and JW3 were annotated and analyzed to identify the major metabolic pathways of carbon, nitrogen, sulfur, and phosphorus based on key genes. JW1^T^ and JW3 can use organic carbon sources (refer to the species description). They harbor key genes of the Entener–Doudoroff pathway, glycolysis pathway, pentose phosphate pathway, and tricarboxylic acid cycle. The genomes of JW1^T^ and JW3 possess an ammonium transporter gene, but they lack genes involved in nitrate reduction, nitrite reduction, nitrogen fixation, nitrification, or anaerobic ammonium oxidation. Thus, both strains can utilize only reduced nitrogen. Genes encoding urease and urea transporter were not detected, suggesting that JW1^T^ and JW3 are incapable of utilizing urea as a C or N source. Both genomes possess a variety of sulfate permease genes involved in assimilatory SO_4_^2+^ reduction. Sulfate can be reduced to sulfide and is subsequently incorporated into amino acids (e.g. cysteine). The genomes of JW1^T^ and JW3 harbor genes encoding low-affinity inorganic phosphate transporter and sodium-dependent phosphate transporter. The presence of alkaline phosphatase genes in both genomes indicates that JW1^T^ and JW3 are capable of utilizing both inorganic and organic forms of phosphorus.

## Conclusion

Strains JW1^T^ and JW3 possess some properties, particularly chemotaxonomic characteristics, that species of the genus *Pseudoalteromonas* all share. On the other hand, JW1^T^ and JW3 could be distinguished from the type strains of their closely related species on the basis of phenotypic differences (e.g., color, NaCl, pH and temperature ranges and optima, arginine dihydrolase, citrate utilization, nitrate reduction, tryptophan deaminase, urease, Voges–Proskauer reaction, carbohydrate utilization, and acid production, Table 1**)**. In addition, strains JW1^T^ and JW3 produce violacein, a potential pharmaceutical agent. On the basis of phylogenetic, genome, and chemotaxonomic data, as well as phenotypic characteristics, strains JW1^T^ and JW3 represent a novel species of the genus *Pseudoalteromonas*, for which the name *Pseudoalteromonas amylolytica* sp. nov. is proposed.

### Description of *Pseudoalteromonas amylolytica* sp. nov

*Pseudoalteromonas amylolytica* (a.my.lo.ly'ti.ca. Gr. n. *amylon*, starch; N.L. adj. *lyticus* -*a* -*um* (from Gr. adj. *lytikos* -*ê* -*on*), able to loosen, able to dissolve; N.L. fem. adj. *amylolytica*, starch dissolving).

Cells are Gram-stain-negative, rod-shaped, 0.7–1.2 μm in width and 1.8–3.0 μm in length. Colonies are violet, circular, convex, smooth and 1–2 mm in diameter after one day of incubation at 30°C on MA. Grow on NaCl-free MB supplemented with 0.5–10% (w/v) NaCl (optimum 1.0–3.0%). pH and temperature ranges for growth are pH 6–10.5 and 20–40°C (optimum at pH 7.5 and 30°C). Require sea salts for growth. No anaerobic growth occurs on MA supplemented with sodium nitrate or sodium nitrite. Produce violacein. Positive for catalase, oxidase, tryptophan deaminase, and Voges–Proskauer reaction. Negative for arginine dihydrolase, citrate utilization, *β*-galactosidase, glucose fermentation, nitrate reduction, lysine and ornithine decarboxylases, indole formation, H_2_S production, and urease. Esculin, gelatin, starch, Tween-20, -40, and -80 are hydrolyzed. The following compounds are utilized as sole carbon and energy sources: *N*-acetyl-glucosamine, l -alanine, l-arginine, d-glucose, l -histidine, l -isoleucine, d-maltose, sodium acetate, sodium propionate, sodium pyruvate, sucrose and d-trehalose. Acid is produced from d-glucose, d-maltose, sucrose, and d-trehalose. Principal fatty acids (> 10%) are summed feature 3 (C_16:1_*ω*7*c* and/or iso-C_15:0_ 2OH), C_18:1_*ω*7*c*, and C_16:0_. Sole respiratory quinone is Q-8. Major polar lipids are phosphatidylethanolamine, phosphatidylglycerol, one unidentified glycolipid, one unidentified aminolipid, and one unidentified phospholipid. In addition, moderate to minor amounts of three unidentified glycolipids, one unidentified aminolipid, one unidentified phospholipid, and two unidentified lipids are present. DNA G+C content is 43.3 mol%.

The type strain, JW1^T^ (= CGMCC 1.15681^T^ = KCTC 52406^T^ = MCCC 1K02162^T^), and additional strain JW3 were isolated from surface seawater.

## Supporting information

S1 Table COG annotations of JW1T, JW3, and related *Pseudoalteromonas* species. (DOCX)

S1 Fig Maximum-likelihood phylogenetic tree based on concatenated sequences of the six housekeeping genes *atpD, gyrB, mreB, recA, rpoD*, and *topA* showing the phylogenetic relationships of JW1T, JW3, and related taxa. The gene sequences were obtained from the genomes, the accession numbers of which are indicated in parentheses. Bootstrap values (>90%) based on 1,000 replications are shown at branch nodes. Bar, 0.05 substitutions per nucleotide position.
(TIF)

S2 Fig **Transmission electron micrographs showing the cell ultrastructure of strains JW1**^**T**^ **(a) and JW3 (b)**. Bar, 0.5 μm.
(TIF)

S3 Fig Absorption profile (a) and mass spectrum (b) of violacein.
(TIF)

S4 Fig **Thin-layer chromatograms after staining with molybdatophosphoric acid, *α*-naphthol reagent, ninhydrin reagent, and molybdenum blue showing the total polar lipid profiles of strains JW1**^**T**^ **(a1-a4), JW3 (b1-b4), and *P*. *byunsanensis* JCM 12483**^**T**^ **(c1-c4).** PE, Phosphatidylethanolamine; PG, phosphatidylglycerol; AL, aminolipid; GL, glycolipid; PL, phospholipid; L, other lipid.
(TIF)
