# Supplementary material for: Physiological and genomic features of a novel violacein-producing bacterium isolated from surface seawater
Source: PLoS One. 2017 Jun 22;12(6):e0179997. doi: 10.1371/journal.pone.0179997 (PMC5481030; doi:10.1371/journal.pone.0179997)
Supplement: S1 Table — (DOCX) [file pone.0179997.s001.docx]

**S1 Table** COG annotations of two isolates and their related *Pseudoalteromonas* species.

Strains/species: 1, strain JW1^T^ (MKJU00000000); 2, strain JW3 (MKJT00000000); 3, *P. byunsanensis* JCM 12483^T^ (MNAN00000000); 4, *P. shioyasakiensis* JCM 18891^T^ (LRUE00000000); 5, *P. arabiensis* JCM 17292^T^ (LRUF00000000); 6, *P. gelatinilytica* NH153^T^ (LRRU00000000).

| COG | Description | 1 | 2 | 3 | 4 | 5 | 6 |
| --- | --- | --- | --- | --- | --- | --- | --- |
| A | RNA processing and modification | 1 | 1 | 1 | 2 | 1 | 2 |
| B | Chromatin structure and dynamics | 3 | 3 | 4 | 3 | 4 | 4 |
| C | Energy production and conversion | 159 | 159 | 162 | 183 | 175 | 171 |
| D | Cell cycle control, cell division, chromosome partitioning | 35 | 35 | 33 | 40 | 40 | 38 |
| E | Amino acid transport and metabolism | 311 | 311 | 303 | 288 | 278 | 291 |
| F | Nucleotide transport and metabolism | 70 | 70 | 67 | 72 | 69 | 69 |
| G | Carbohydrate transport and metabolism | 134 | 134 | 127 | 160 | 148 | 152 |
| H | Coenzyme transport and metabolism | 158 | 158 | 150 | 138 | 151 | 133 |
| I | Lipid transport and metabolism | 112 | 112 | 104 | 110 | 116 | 111 |
| J | Translation, ribosomal structure and biogenesis | 203 | 204 | 203 | 191 | 191 | 190 |
| K | Transcription | 256 | 256 | 238 | 280 | 261 | 266 |
| L | Replication, recombination and repair | 177 | 176 | 150 | 166 | 152 | 169 |
| M | Cell wall/membrane/envelope biogenesis | 206 | 207 | 209 | 247 | 216 | 244 |
| N | Cell motility | 119 | 119 | 139 | 120 | 147 | 126 |
| O | Posttranslational modification, protein turnover, chaperones | 151 | 151 | 145 | 158 | 152 | 162 |
| P | Inorganic ion transport and metabolism | 172 | 172 | 158 | 219 | 207 | 204 |
| Q | Secondary metabolites biosynthesis, transport and catabolism | 109 | 109 | 85 | 67 | 66 | 64 |
| R | General function prediction only | 403 | 403 | 386 | 393 | 384 | 394 |
| S | Function unknown | 282 | 283 | 272 | 327 | 322 | 339 |
| T | Signal transduction mechanisms | 397 | 397 | 366 | 440 | 390 | 432 |
| U | Intracellular trafficking, secretion, and vesicular transport | 105 | 105 | 109 | 114 | 122 | 112 |
| V | Defense mechanisms | 84 | 84 | 76 | 67 | 67 | 68 |
| Z | Cytoskeleton | 0 | 0 | 0 | 1 | 0 | 2 |
|  | Total features in COGs | 3647 | 3649 | 3487 | 3786 | 3659 | 3743 |
