# Supplementary figures and images for: Physiological and genomic features of a novel violacein-producing bacterium isolated from surface seawater
Source: PLoS One. 2017 Jun 22;12(6):e0179997. doi: 10.1371/journal.pone.0179997 (PMC5481030; doi:10.1371/journal.pone.0179997)

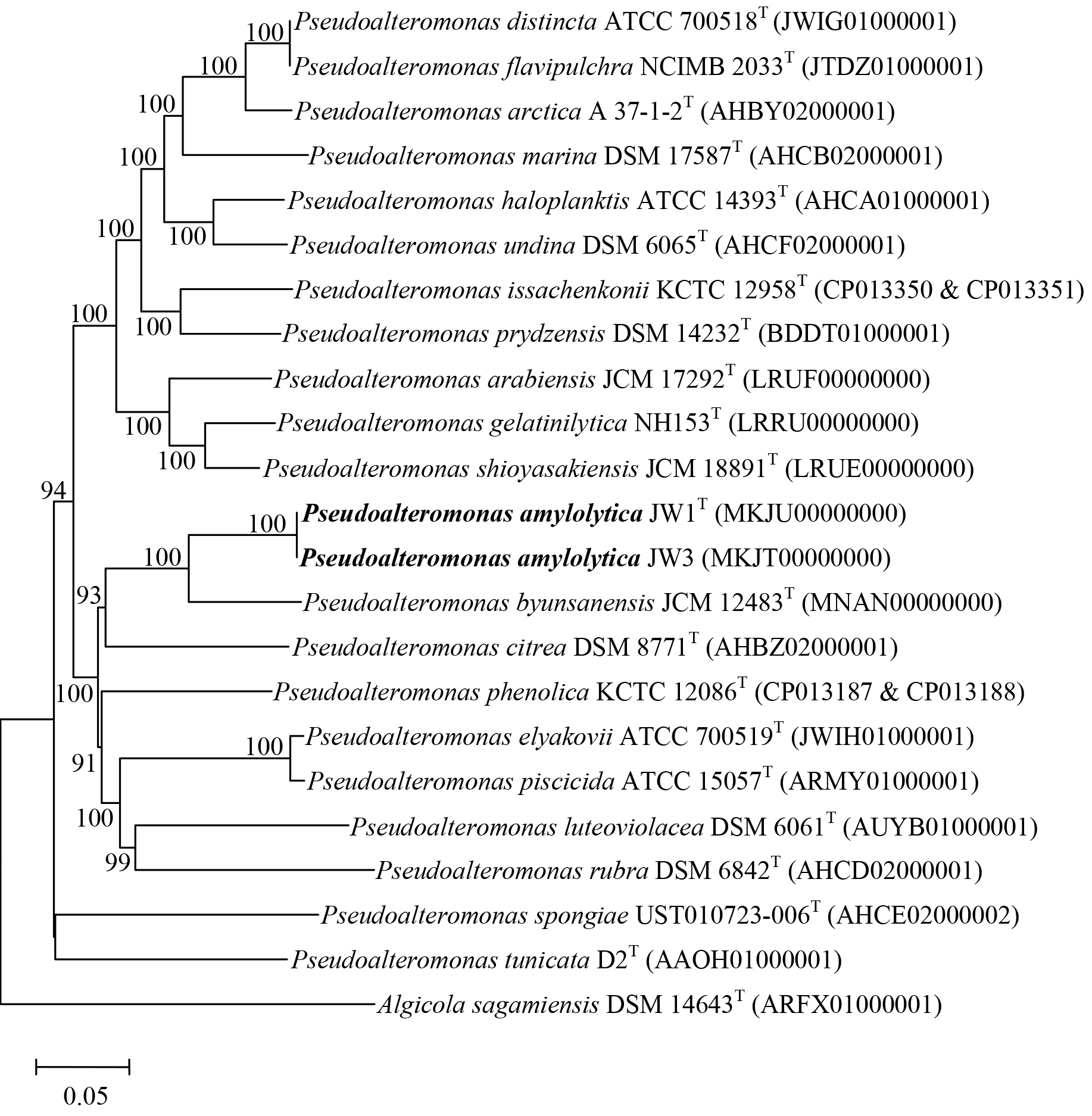

Supplement: S1 Fig — The gene sequences were obtained from the genomes, the accession numbers of which are indicated in parentheses. Bootstrap values (>90%) based on 1,000 replications are shown at branch nodes. Bar, 0.05 substitutions per nucleotide position. (TIF) [file pone.0179997.s002.tif]

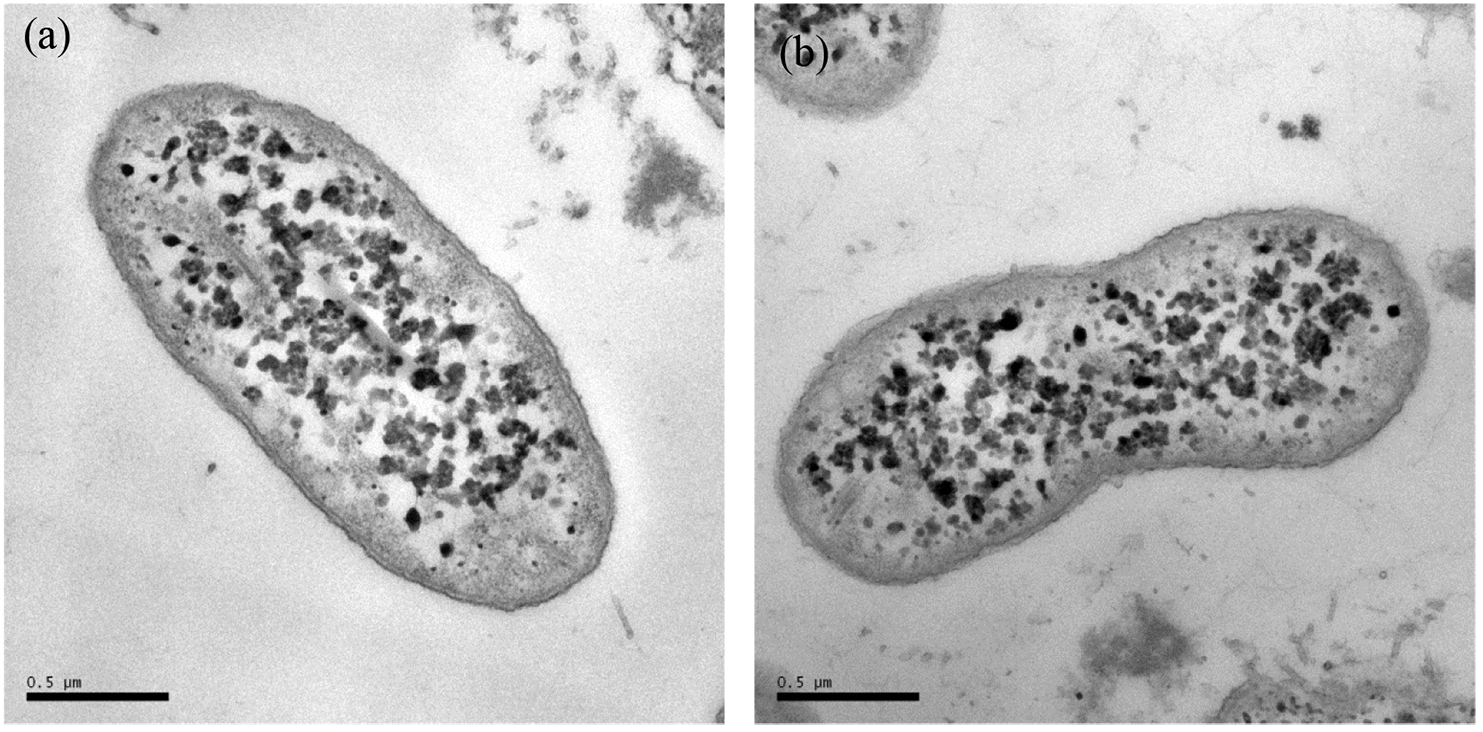

Supplement: S2 Fig — Transmission electron micrographs showing the cell ultrastructure of strains JW1T (a) and JW3 (b). Bar, 0.5 μm. (TIF) [file pone.0179997.s003.tif]

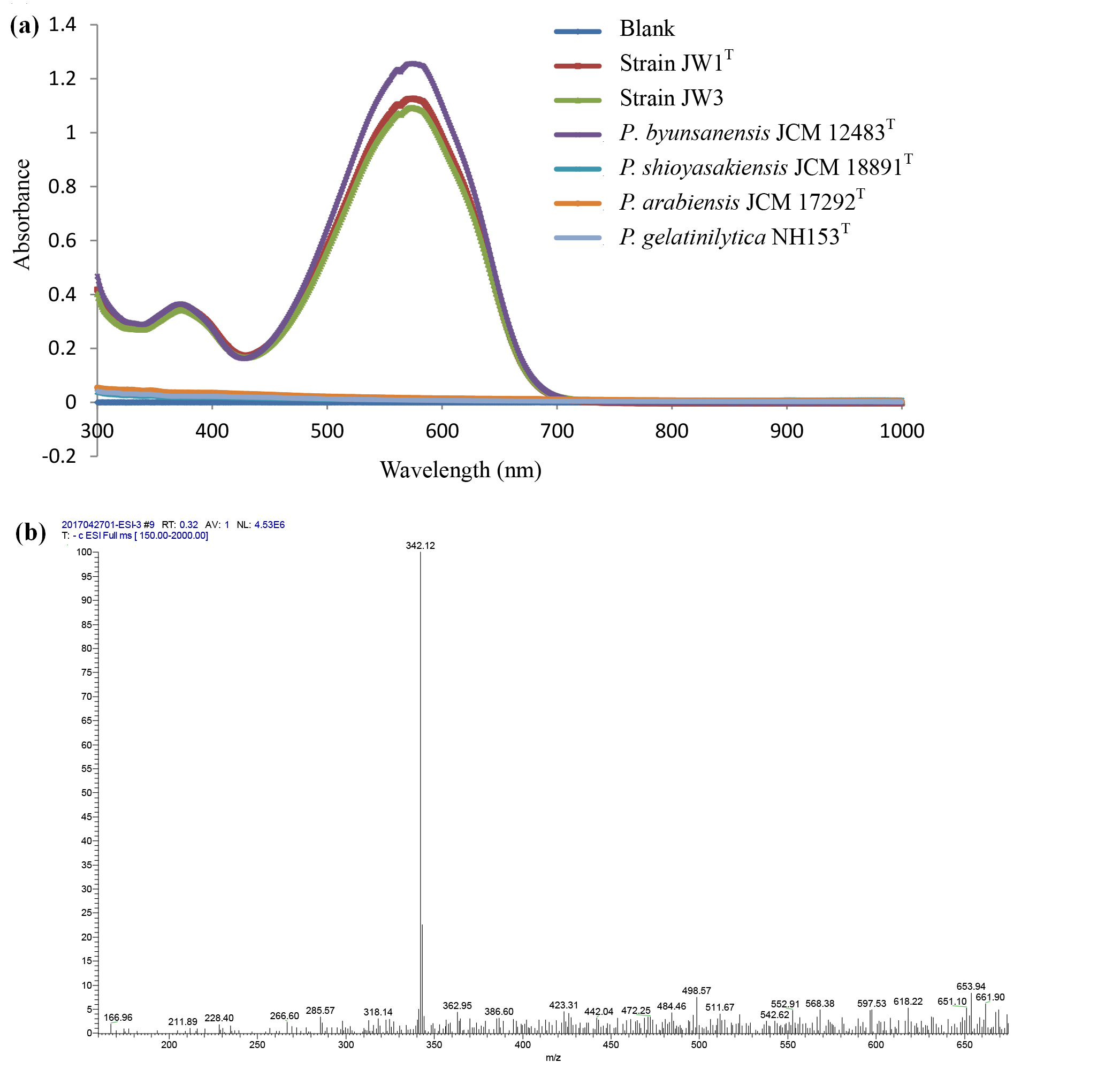

Supplement: S3 Fig — Absorption profile (a) and mass spectrum (b) of violacein. (TIF) [file pone.0179997.s004.tif]

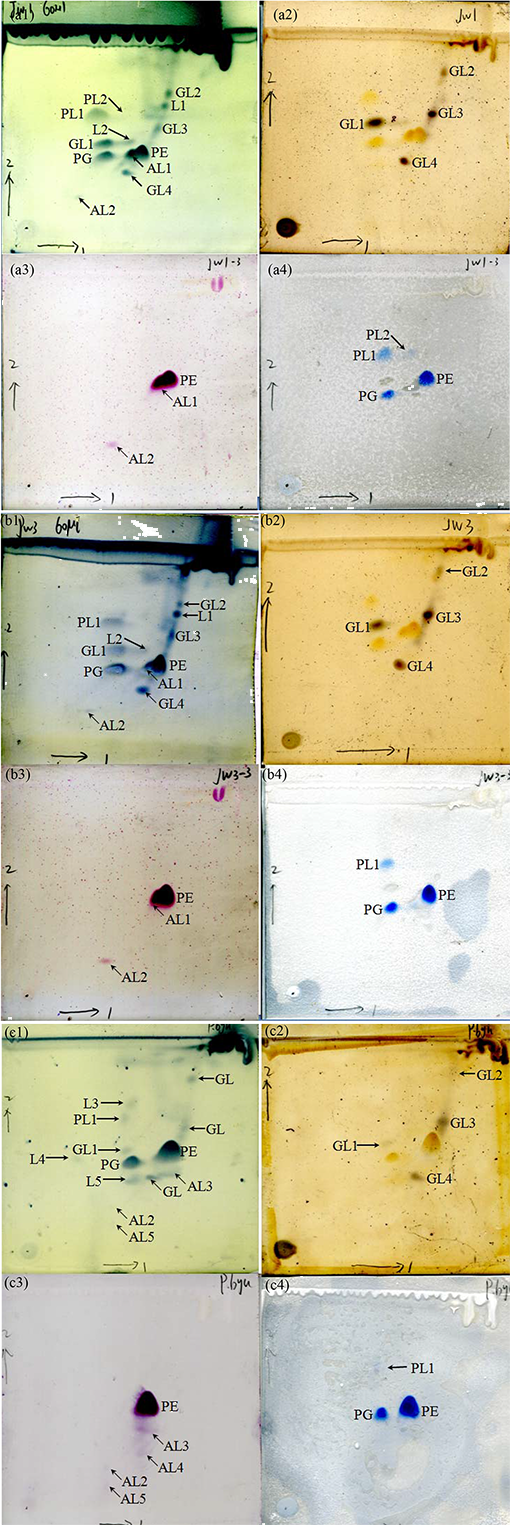

Supplement: S4 Fig — Thin-layer chromatograms after staining with molybdatophosphoric acid, α-naphthol reagent, ninhydrin reagent, and molybdenum blue showing the total polar lipid profiles of strains JW1T (a1-a4), JW3 (b1-b4), and P. byunsanensis JCM 12483T (c1-c4). PE, Phosphatidylethanolamine; PG, phosphatidylglycerol; AL, aminolipid; GL, glycolipid; PL, phospholipid; L, other lipid. (TIF) [file pone.0179997.s005.tif]
